# Taurine: A Maternally Derived Nutrient Linking Mother and Offspring

**DOI:** 10.3390/metabo12030228

**Published:** 2022-03-05

**Authors:** Shiro Tochitani

**Affiliations:** 1 Division of Health Science, Graduate School of Health Science, Suzuka University of Medical Science, Suzuka 513-8670, Japan; tochitani.shiro@gmail.com; Tel.: +81-59-373-7069; 2 Department of Radiological Technology, Faculty of Health Science, Suzuka University of Medical Science, Suzuka 513-8670, Japan; 3 Center for Preventive Medical Sciences, Chiba University, Chiba 263-8522, Japan; 4 Department of Neurophysiology, Hamamatsu University School of Medicine, Hamamatsu 431-3192, Japan

**Keywords:** 2-aminoethanesulfonic acid, developmental programming, GABA_A_ receptors, glycine receptors, neural progenitor, neural stem cell, perinatal nutrition, placental transfer, obesity, taurine transporter

## Abstract

Mammals can obtain taurine from food and synthesize it from sulfur-containing amino acids. Mammalian fetuses and infants have little ability to synthesize taurine. Therefore, they are dependent on taurine given from mothers either via the placenta or via breast milk. Many lines of evidence demonstrate that maternally derived taurine is essential for offspring development, shaping various traits in adults. Various environmental factors, including maternal obesity, preeclampsia, and undernutrition, can affect the efficacy of taurine transfer via either the placenta or breast milk. Thus, maternally derived taurine during the perinatal period can influence the offspring’s development and even determine health and disease later in life. In this review, I will discuss the biological function of taurine during development and the regulatory mechanisms of taurine transport from mother to offspring. I also refer to the possible environmental factors affecting taurine functions in mother-offspring bonding during perinatal periods. The possible functions of taurine as a determinant of gut microbiota and in the context of the Developmental Origins of Health and Disease (DOHaD) hypothesis will also be discussed.

## 1. Introduction

A significant development in the evolution of mammals is placentation, intrauterine development of the fetus, and extensive care after birth that improves infant survival to a reproductive age [1]. Only female mammals have the ability to provide prenatal resources through the placenta and produce milk for postnatal development [2]. Not surprisingly, females form their strongest social bonds with their offspring [1]. In addition, offspring development can be affected by early mother–offspring relationships [3]. Thus, it is essential to understand the nature of mother–offspring bonding during pregnancy and the postpartum period and the factors that affect bonding [3].

Taurine (2-aminoethanesulfonic acid) is a sulfur-containing organic acid with various biological functions, including membrane stabilization, cell volume regulation, mitochondrial protein translocation, anti-oxidative activity, and modulation of intracellular calcium levels [4,5]. In addition, taurine structurally resembles neurotransmitter γ-aminobutyric acid (GABA) and glycine and interacts with both GABA_A_ and glycine receptors to induce chloride currents in neuronal cells [5,6,7].

**Figure 1:**
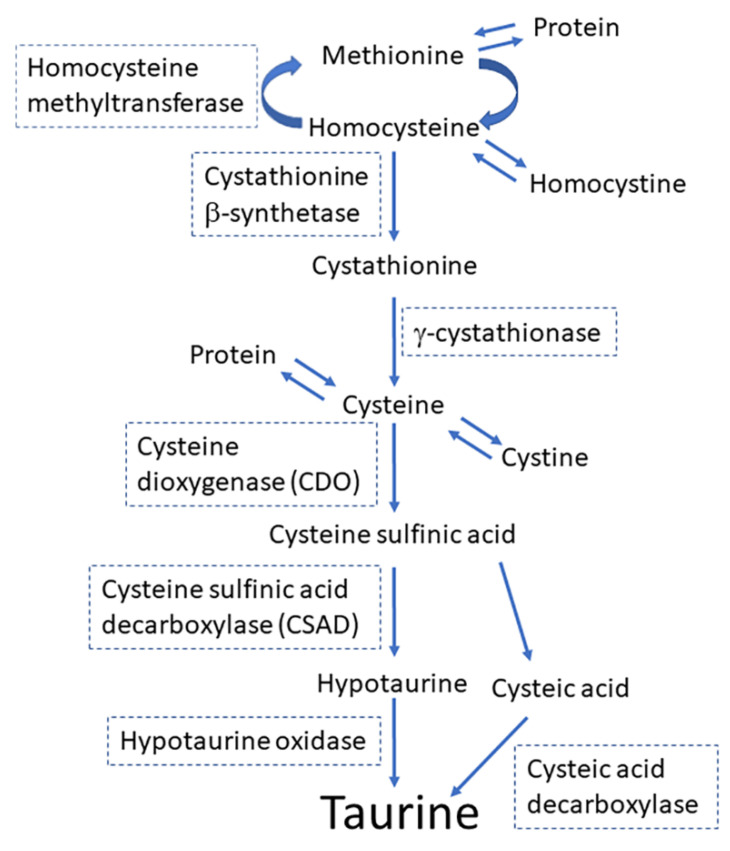
The principal pathway for the synthesis of taurine from methionine and cysteine.

In mammals, adults synthesize taurine in the liver from methionine/cystine, although fetuses and infants have limited ability to synthesize taurine because they have limited levels of γ-cystathionase and cysteine sulfinic acid (CSAD) in livers and brains (Figure 1) [8]. Fetuses and infants depend on the taurine supplied by mothers via the placenta or breast milk [5,9]. Taurine is a principal constituent of the amino acid pool in the milk in many species, including humans, chimpanzees, baboons, rhesus monkeys, Java monkeys, sheep, and rats [10,11]. Taurine has the second highest concentration in breast milk after glutamate in these species [10]. Notably, in cats, in which taurine deficiency during early development leads to severe morphological developments of the retina and cerebellum [12,13], the concentration of taurine in milk is very high (2.87 M), being second to that in gerbil (5.95 M) [8]. Taurine is a molecule that links the mother with the offspring. In this review, I provide a comprehensive overview of the functions of taurine in offspring development, the regulatory mechanisms of taurine transport from mother to offspring, and the outcomes of taurine depletion during development.

## 2. Transport of Taurine from Mothers to Offspring

### 2.1. Cloning of a Taurine Transporter and Its Function

The molecular system for taurine transport has been revealed by conducting various physiological analyses. The taurine transport system is energized by a Na^+^ gradient and requires Cl^−^, and its activity is inhibited by Ca^2+^/diacylglycerol-dependent protein kinase (PKC) [14]. In addition, taurine efflux is stimulated by hypoosmotic conditions [15]. Cloning cDNAs for taurine transporter (TauT) revealed an aspect of the molecular basis for taurine transport with these physiological properties. The cDNAs encoding for the taurine transporter (TauT) were first cloned using sequence similarities to glycine, GABA, or other neurotransmitter transporters from Madin–Darby canine kidney cells and mouse and rat brains [16,17,18]. Then, human TauT was cloned from the human placenta using a similar approach [19]. The Na^+^/Cl^−^/taurine stoichiometry of the cloned TauT was 2:1:1, and the transporter was specific for taurine and other β-amino acids with a high affinity for taurine [19].

### 2.2. Mechanisms Regulating the Activity of the Taurine Transporter

Taurine transporter activity was suppressed by exposure to taurine in JAR choriocarcinoma cells by both transcriptional and posttranscriptional mechanisms [20]. Another group also reported that TauT activity was downregulated by taurine itself in human intestinal Caco-2 cells, and this adaptive regulation was also induced by hypotaurine and β-alanine [21].

As for transcriptional regulatory mechanisms, TauT gene expression was elevated under high nitric oxide (NO) level conditions in retinal pigment epithelial cells [22]. Wilms tumor suppressor gene (WT1) enhances the transcription of TauT in human embryonic kidney 293 cells in a concentration-dependent manner [23]. Taurine transporter activity and the expression of TauT mRNA were upregulated by hypertonicity in mouse 3T3-L1 adipocytes [24]. Exposure to various prooxidants, including H_2_O_2,_ induced the promoter activity of TauT in human retinal pigment epithelial cells [25]. Myoblast determination protein 1 (often abbreviated as MyoD) overexpression induced TauT promoter activity in mouse C3H10T1/2 fibroblasts [26]. Glucose reduced TauT mRNA and protein expression in a concentration-dependent manner in human Schwann cells [27].

The acute regulation of TauT activity involves a shift in pH and membrane potential (uptake is reduced following acidification of the extracellular medium and depolarization of the plasma membrane) and phosphorylation/dephosphorylation of TauT [28]. The cloned retinal TauT expressed in Xenopus oocytes is inhibited by the activation of cAMP-dependent protein kinase (PKA) and PKC [29]. Taurine uptake was downregulated by applying a PKC activator in the rat conditionally immortalized STB cell line (TR-TBT cells) [30]. Taurine uptake was inhibited, and efflux was accelerated under calcium-free conditions in TR-TBT cells [30]. Conditionally immortalized rat brain capillary endothelial cells (TR-BBB13), an in vitro model of the blood–brain barrier (BBB), showed an enhanced uptake of taurine by exposure to both tumor necrosis factor α (TNF-α) and a hypertonic condition [31]. Mochizuki et al. also showed that TNF-α markedly enhanced TauT activity in human intestinal Caco-2 cells. They proposed that the enhanced transport of taurine into cells underlies the cellular response against intestinal inflammation [32]. They further showed that nuclear factor κB (NF-κB) was involved in the signaling mechanism for the upregulation of taurine uptake and TauT mRNA expression induced by TNF-α [33]. Another group reported that TNF-α, lipopolysaccharide (LPS), and diethyl maleate (DEM) significantly increase taurine uptake, but H_2_O_2_ and an NO donor decreased taurine uptake in the cells [30]. Osmolarity also matters in the regulation of TauT activity. Upregulation of TauT mRNA transcription and TauT activity by hypertonic conditions in Caco-2 cells depends on calcium/calmodulin-dependent protein kinase II (Ca^2+^/CaM kinase II) [34]. Cell swelling induced by hypoosmotic conditions results in reactive oxygen species production, which accounts for reduced taurine uptake under low-sodium/hypo-osmotic conditions by the direct modulation of TauT in NIH3T3 mouse fibroblasts [35]. Elevated levels of cortisol and IGF-1 also upregulate taurine transport in L6, a rat skeletal muscle cell line [36].

In summary, taurine itself downregulates TauT activity by transcriptional and posttranscriptional mechanisms. NO, hypertonicity, prooxidants, and glucose can upregulate TauT mRNA transcription. The factors that acutely enhance taurine transport by TauT include hypertonic condition, TNF-α, cortisol, LPS, and DEM. The factors that acutely inhibit taurine’s transport by TauT include high glucose levels, hypoosmotic condition, and H_2_O_2_. In addition, NO enhances TauT activity in retinal pigment epithelial cells, while NO downregulates taurine uptake in TR-TBT cells.

### 2.3. Transfer of Taurine to the Fetus via the Placenta

#### 2.3.1. Machinery for Taurine Transport in the Placenta

Sturman et al. have shown that [^35^S]-labeled taurine, injected intraperitoneally into pregnant rats, can be delivered to both the brain and liver of the fetus, suggesting that maternal taurine can be transmitted to the fetus via the placenta [37]. Taurine is ranked as the most abundant free amino acid in the human placenta [38]. The human placenta transports taurine from the mother to the fetus by an active process because the taurine concentration in fetal blood is higher than in maternal blood [14]. Hibbard et al. demonstrated that the perfused human placenta can achieve and maintain that the ratio of fetal to maternal taurine concentration is, on average, 1.38:1, suggesting that taurine is transported in the placenta by an active transport mechanism [39]. Furthermore, the placental tissue concentration of taurine is 100 to 150-fold higher than that in fetal and maternal circulations [14]. Syncytiotrophoblast (STB) cells in the placenta possess an active transport system for taurine [40]. Kulanthaivel et al. found that the JAR human placental choriocarcinoma cell line can transport taurine, concentrating over 1000-fold inside the cell from the medium [14]. STB represents the primary barrier for transferring nutrients from the mother to the fetus in the human placenta. Maternal blood accumulates in the intervillous space and bathes the microvillous membrane (MVM). The basal plasma membrane (BM) of the STB is oriented toward fetal circulation. Transporters transferring amino acids, glucose, and fatty acids are expressed in both plasma membranes of the STB [41]. Taurine transport to the fetus also includes its uptake from maternal blood by transfer across the MVM of STB and, subsequently, they are transported to the fetus across BM [42]. The tissue concentrations of taurine in the human placenta are 100 to 200-fold higher than that in maternal blood, indicating the presence of an efficient active transport of taurine in the MVM [42]. Norberg et al. demonstrated that the activity of Na^+^-dependent taurine transport, which is based on TauT, in BM was only 6% of that in MVM. In contrast, Na^+^-independent transport activities also exist both in MVM and BM, although the details for Na^+^-independent taurine transport remain to be elucidated [42]. The highly polarized distribution of Na^+^/taurine cotransporters in the MVM in conjunction with similar Na^+^-independent transport rates for taurine in MVM and BM provides the basis for net taurine flux between the mother and the fetus [42].

As for species’ differences, the expression levels of TauT proteins were slightly lower in plasma membrane vesicles isolated from the mouse placenta than in human MVM, suggesting that the relative significance of placental taurine transport for fetal development is lower in mice than in humans [43]. However, the importance of the system β-amino acid transporter, encoded by the TauT gene, in promoting normal growth is exemplified by the observation that mice homozygous for the deletion of the TauT gene (taut−/−) are significantly smaller than their wild-type siblings and do not exhibit catch-up growth [43,44].

Amniotic fluid (AF) contains growth factors and principal nutrients that facilitate fetal growth and provide mechanical cushioning [45]. AF contains taurine, which is found in greater quantities in AF than in maternal serum. At the same time, most other amino acids have lower concentrations in AF than in maternal and fetal blood, indicating the activity of an unidentified mechanism by which taurine is enriched in AF [45].

In summary, the placental tissues concentrate taurine efficiently and transfer taurine to fetal circulation by Na^+^-dependent taurine transport, which is based on TauT activity. Although the detailed mechanisms remain to be elucidated, taurine is also enriched in AF to a great extent. These observations suggest that taurine is essential in mother–fetus relationships, and taurine transport has to be adequately regulated during pregnancy.

#### 2.3.2. Environmental Factors Affecting Placental Taurine Transport

In intrauterine growth restriction (IUGR), MVM Na^+^-dependent taurine transport was found to be reduced by up to 34%, whereas Na^+^-independent uptake was unaltered, suggesting that the impairment in placental taurine transport can be a causal factor of IUGR [42]. Roos et al. found that MVM TauT expression was unaltered in IUGR, whereas NO release downregulated placental TauT activity [46]. Ditchfield et al. showed that the placental TauT activity was significantly lower in obese women (body mass index (BMI) > 30) than women of ideal weight [47]. TauT activity in STB, measured in fragments of placental tissue, was negatively correlated with maternal BMI [48]. STB TauT activity was significantly lower in preeclampsia (PE) than in normal pregnancy [48]. In baboons, maternal nutrient restriction reduced fetal weight by 13%, decreased taurine concentration in fetal serum, and decreased TauT protein expression in MVM [49].

Preeclampsia is associated with NO signaling [50]. Therefore, the observation that PE is associated with reduced TauT activity is consistent with the observation that NO decreased taurine uptake in immortalized STB cell line TR-TBT [30]. High glucose levels inhibit TauT activity [27]. It can be inferred that PE and obesity are associated with reduced placental TauT activity because high blood glucose levels are well associated with both physiological conditions [51,52]. It is well known that, in obesity, adipose tissues release inflammatory mediators such as TNF-α and interleukin-6, predisposing the system to a pro-inflammatory state and to oxidative stress [47,53]. Severe social stress and chronic hypertension can act in combination to increase the risk of PE [54]. However, as described, TNF-α was observed to enhance taurine transport by TauT in the study using TR-TBT cells [30], and cortisol enhances TauT activity in a muscle cell line [36]. These in vitro observations are not apparently consistent with reduced TauT activity in the placenta in both obesity and PE. The detailed mechanisms for the downregulation of placental TauT activity in obesity and PE need further investigation.

Maternal environmental factors including obesity, PE, and nutrient restriction can suppress placental taurine transport. Environmental factors affecting placental taurine transfer can finally influence fetal development. These environmental factors are well known for their associations with undesirable developmental outcomes. For example, maternal obesity can be a risk factor for various mental disorders by affecting the development of the brains and neural circuits of the offspring [55]. Obstetric complications, including PE and nutrient deficiencies, also increase the risks for psychiatric disorders by inducing abnormal brain development during prenatal and postnatal periods [56]. Taurine might be a critical factor in these causal relationships.

### 2.4. Transfer of Taurine to Offspring during Postnatal Care

#### 2.4.1. Transfer of Taurine to Offspring via Breast Milk

Exclusive breastfeeding, the practice of only providing breast milk for the first 6 months of an infant’s life (no other food or water) provides essential, irreplaceable nutrition for a child’s growth and development [57]. Taurine is a significant component of free amino acids in the milk of many species and is second in concentration to glutamate [10]. Taurine concentrations in chimpanzees, rhesus monkeys, sheep, and rats were found to be the highest during the first days of lactation, and it decreased to a particular constant concentration after the first week [10]. Sturman et al., by injecting [^35^S]taurine intraperitoneally into lactating dams after parturition, demonstrated that taurine was transferred to pups via milk and accumulated in brains to a greater extent than in livers [58].

Milk production in the mammary gland mainly depends on milk synthesis and the proliferation abilities of mammary epithelial cells (MECs) [59,60]. Milk synthesis is the combined result of several intracellular processes within MECs [61]. Proteins synthesized in the endoplasmic reticulum of MECs are packaged into vesicles within the Golgi apparatus and then released by exocytosis. Some vesicles containing other proteins, such as IgA, are transported across the apical membrane. Some monosaccharides, sodium, potassium, chloride, and water can directly pass through the apical membrane [61]. Under the influence of progesterone and prolactin, MECs differentiate into the lobuloalveolar complex: a single layer of polarized MECs surrounding a lumen connected to the central duct system [61]. With the fall of progesterone at the end of pregnancy, when tight junctions form between MECs, milk comes to be contained within the lumen of the lobuloalveolar complex, and it becomes available for secretion [61].

Taurine transporters are expressed in mammary glands [62]. Aleman et al. showed that TauT mRNA was abundant during pregnancy in rat mammary glands. However, the expression levels decreased after the onset of lactation and stabilized around the levels observed in virgin rats [62]. Another study in mice reported lower transcription levels of TauT during lactation compared to the levels during pregnancy [63]. As shown in Figure 1, taurine was synthesized from cysteine via oxidation of cysteine to cysteine sulfinic acid by cysteine dioxygenase (CDO), followed by the decarboxylation of cysteine sulfinic acid to hypotaurine, a precursor of taurine, which is catalyzed by CSAD [64]. The mRNA transcription level for CSAD, a rate-limiting enzyme for taurine synthesis, was higher during early lactation (day 1 and 6 of lactation) than in the later lactational stage (day 14) in rat mammary glands [65]. CSAD mRNA was observed to be expressed in MECs in rat mammary glands [65]. The expression of CDO proteins was observed, preferentially, in the ductal cells of pregnant rats but not in other MECs or the ductal cells of nonpregnant rats [64]. In rats, milk taurine concentrations were the highest in early lactation, right after birth, and declined rapidly after the onset of lactation [64,66]. The expression levels of CDO proteins in the mammary tissue increased, and CSAD protein expression levels declined only slightly throughout lactation [64]. These results suggest that, in addition to taurine transported by MECs from maternal blood, a significant amount of taurine contained in mother’s milk may be synthesized de novo in MECs [62].

#### 2.4.2. Taurine in Infant Formula

As described, in the liver and brains of human fetuses and newborn infants, the levels of taurine biosynthesis are scant due to the limited activities of γ-cystathionase and CSAD [8,67,68]. In addition, human milk contained a large amount of taurine (450 to 500 mg/L), which resulted in the widely accepted notion that taurine functions as semi-essential amino acids [69]. These observations and widely accepted notions have supported the decision by the US Food and Drug Administration to permit adding taurine to purified infant formulas [69]. Analyses on free amino acids included in the commercially available formulas in the 2000s in Europe revealed that the reconstituted infant formulae contained a mean of 4.0 mg taurine/100 mL, and the reconstituted follow-up milk contained 1.8 mg taurine/100 mL [70]. The mean content of taurine found in infant formulae was similar to that found in human milk [70]. A recent systematic review found a complete lack of scientific evidence with regards to the developmental benefits of adding taurine to infant formula [71]. Therefore, further clinical studies are necessary for the evaluation of the beneficial effects of taurine in infant formulas.

#### 2.4.3. Environmental Factors Affecting Taurine Transfer during Postnatal Period via Breast Milk

Intraperitoneal injection of taurine into lactating mouse dams did not affect taurine concentration in breast milk, but the injection of β-alanine significantly decreased taurine concentrations [72]. β-alanine administration caused a significant reduction in taurine concentration in the brains of offspring, and the taurine concentration in the brain was negatively correlated with the total distance traveled in the open field test at postnatal day 15, suggesting that a decreased concentration of taurine in the mother’s milk can alter offspring behavior [72]. Interestingly, restraint stress in lactating mice caused an increase in the concentration of taurine and cystathionine in milk. However, restraint stress did not alter their concentration in the maternal plasma, liver, and mammary glands [73]. The ratio of taurine concentration in breast milk to its concentration in maternal plasma was significantly increased in the restraint stress group, suggesting that maternal stress promoted taurine transportation from maternal blood into milk [73]. Although the detailed molecular mechanisms underlying this phenomenon remain to be elucidated, the enhanced transport of taurine into milk under maternal stress conditions may be associated with the upregulation of the activity of TauT by the stress hormone, cortisol, at the cellular level as described above [36].

Summarizing the environmental factors relevant to taurine transport to offspring via mother’s milk, excessive β-alanine administration can be a factor that decreases taurine concentration in breast milk, while maternal stress may enhance taurine transport to milk.

## 3. Biological Functions of Taurine during Development

### 3.1. Developmental Outcomes of Taurine-Depletion

The crucial observations related to the biological outcomes induced by taurine depletion were obtained in cats and monkeys. Cats fed a casein diet exhibited retinal degeneration [12]. Plasma amino acid profiles demonstrated an essential absence of plasma taurine in taurine-depleted cats. In contrast, concentrations of other amino acids, including methionine and cystine (taurine precursors), were comparable to the control values [12]. Adult female cats fed a defined taurine-free diet exhibited a low fertility rate and suffered retinal degeneration [74]. Except for taurine content, the maternal milk of the taurine-depleted mothers remained unchanged [74]. The concentration of taurine in the milk from taurine-deprived feline mothers was reduced to 5.9% of that from taurine-supplemented dams [5,13]. The surviving offspring from the taurine-depleted mothers exhibited a variety of neurological abnormalities and reduced concentrations of taurine in body tissues and fluids [74]. Neuringer and Sturman fed rhesus monkeys a taurine-free, soy protein-based infant formula from birth to 3 months of age, which resulted in impaired visual acuity [75]. Hayes and colleagues raised infant monkeys from birth with soybean infant milk formula lacking taurine and found significant growth depression, suggesting that dietary taurine is essential for infant development [76].

TauT knockout (KO) mice exhibited reduced fertility and retinal degeneration [77]. In several other studies, TauT KO mice exhibited a deficiency in myocardial and skeletal muscle taurine content compared to their wild-type littermates [44,78,79]. The TauT KO heart was characterized by a reduction in ventricular wall thickness and cardiac atrophy accompanied by smaller cardiomyocytes [78]. The skeletal muscles of TauT KO mice also exhibited decreased cell volume, structural defects, and a reduction in exercise endurance [78,79,80,81]. The expression of molecules related to osmotic stress was upregulated in both the heart and skeletal muscle of the TauT KO mice [78]. Tissue taurine depletion in TauT KO mice shortened the lifespan and accelerated the histological and functional defects of skeletal muscles, probably caused by cellular senescence [81]. Unfolded protein response was more often observed in TauT KO muscles than in control muscles, suggesting that taurine depletion causes an accumulation of protein misfolding, which accelerates cellular aging [81].

### 3.2. Possible Biological Mechanisms Underlying the Function of Taurine during Development

#### 3.2.1. Taurine as Agonist for Receptors

Taurine is one of the most plentiful free amino acids in the developing central nervous system [82,83]. Extracellular taurine stimulates neurons and neuronal progenitor cells mainly by way of GABA_A_ with affinities for specific receptor subtypes [83,84,85]. Taurine functions also as an agonist for GABA_B_ receptors [84,86,87]. Taurine also functions as a partial agonist of glycine receptors [84]. Furthermore, neurons undergo specific maturation steps during brain development, including neurogenesis, neuronal migration, and neuronal anatomical and functional maturation, where taurine can function as extrinsic instructive signals for the neural progenitors (NPs) and the newly generated neurons in the developing central nervous system (CNS) [84].

During neurogenesis, NPs in a specific region of the CNS produce specific types of neurons in a defined order with precise timing throughout the course of CNS development [88,89]. The temporal and spatial specifications of NPs are essential for CNS histogenesis [89]. Temporal changes in NP differentiation are driven by a combination of intrinsic cellular properties and extracellular signals from the environment of the developing brain [90]. Neural progenitors express GABA_A_ receptors in the developing cortex [91,92]. In the mouse developing cerebral cortex, Tochitani et al. demonstrated that taurine functions as an agonist for GABA_A_ receptors to instruct temporal specifications of the NPs producing excitatory glutamatergic neurons [93]. Neural progenitors respond to taurine before embryonic day 13 (E13) through GABA_A_ receptors, while NPs respond to GABA only after E13 [93]. Furthermore, the endogenous sources for non-synaptic GABA, such as the meninges and choroid plexus, are not fully developed before E13 in the developing cortex [94]. The taurine concentration was almost 500-times higher than GABA concentrations in the telencephalon at E13 [93]. GABA_A_ antagonist administration to pregnant dams on E10–12, at which taurine functions as a principal endogenous agonist for GABA_A_ receptors, resulted in the development of autistic behavior in offsprings [93]. These results not only demonstrate that taurine plays a vital role as an agonist for GABA_A_ receptors but also that disruptions of taurine-GABA_A_ receptor interaction can result in neurodevelopmental disorders.

Aberrant layer formation in the cerebrum and visual cortex was observed in the surviving kitten from taurine-depleted dams, suggesting that taurine plays a role in neuronal migration during brain development [13,95]. Behar et al. have shown that taurine and GABA_B_ receptors mediate motility signals for the radial migration of newly generated neurons from the ventricular zone to the cortical plate in the developing rat cortex [96]. Recently, Furukawa et al. found that radial migration was not affected in a homozygous Glutamic acid decarboxylase 67 (GAD-67) deficient mouse, in which GABA content was reduced to 12.7% of the wild-type level [84,97]. However, the inhibition of GABA_A_ receptors accelerated radial migration in this GAD-67-deficient mouse to a similar extent as in the wild-type animals, indicating that GABA is not required as an endogenous agonist for GABA_A_ receptors regulating migration [84,97]. Furthermore, they observed that the radial migration of newly generated neurons was accelerated by maternal administration of D-cysteine sulfinic acid, a competitive inhibitor of taurine production, in both wild-type and homozygous GAD-67 deficient mice, suggesting that taurine is indeed a major endogenous modulator of radial migration [84,97]. Glycine receptors also participate in regulating the radial migration of newly generated neurons. Nimmervoll et al. showed that the application of strychnine, an antagonist of glycine receptors, retarded radial migration [98]. The application of strychnine without glycine did not cause any effects, suggesting that endogenous glycine does not induce the phenomenon. However, it remains unelucidated whether taurine can function as an endogenous agonist for glycine receptors to regulate the migration of newly generated neurons [98].

**Figure 2:**
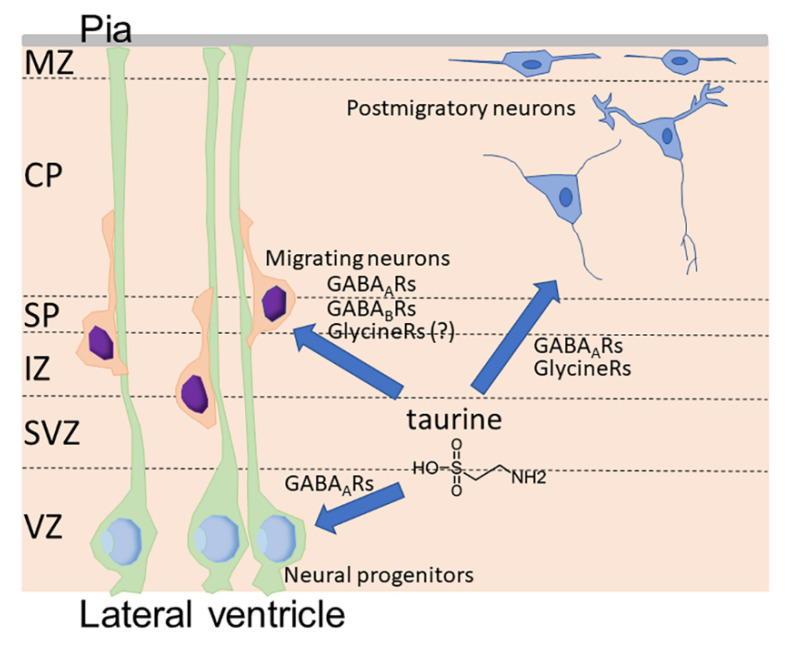
Functions of taurine as endogenous agonists for GABA_A_ receptors (GABA_A_Rs), GABA_B_ receptors (GABA_B_Rs), and glycine receptors (Glycine Rs) in the developing cortex. CP, cortical plate; IZ, intermediate zone; MZ, marginal zone; Pia, pia mater; SP, subplate; SVZ, subventricular zone; VZ, ventricular zone.

In the developing cortex, taurine accumulates in the cells in specific layers called the marginal zone (MZ) and subplate (Figure 2) [93,97]. A few cells in the cortical plate were also observed to contain high concentrations of taurine [93]. Extracellular taurine is released from cells through volume-regulated anion channels [97]. The MZ is the most superficial layer composed of the early generated neurons in the developing cortex (Figure 2). Cajal–Retzius cells in the MZ express glycine receptors and the activation of glycine receptors in the MZ results in membrane depolarization [99,100]. Qian et al. showed that a single electrical stimulation in MZ leads to the radial propagation of membrane depolarization in MZ, which can be suppressed by either GABA_A_ or glycine receptor antagonists [101]. Qian et al. further showed that electrical stimulations induced the release of taurine and GABA, but not glycine, from the cells in MZ, suggesting that the activity-dependent release of taurine by cells in MZ mediates excitatory neurotransmission via GABA_A_ and glycine receptors [101]. Flint et al. showed that the immature postmitotic neurons in the intermediate zone and the cortical plate in the developing cortex express functional glycine receptors, which are excitatory, while neural progenitors in the ventricular zone do not express functional glycine receptors [102]. Flint et al. also found that the extracellular accumulation of taurine induced by applications of guanidinoethylsulfinate, an inhibitor of sodium-taurine cotransport, produced large inward currents that were reversibly blocked by strychnine, an inhibitor of glycine receptors, suggesting that taurine functions as an endogenous agonist for glycine receptors in the immature postmitotic neurons in the developing cortex [102].

As previously mentioned, AF contains taurine in greater concentrations than in maternal serum. The AF trapped in the neural tube serves as the initial cerebrospinal fluid (CSF) during neural tube closure [103,104]. The cells facing the lateral ventricle were also rich in taurine in addition to the cells in the mantle zone and subplate, especially in the early phase of cortical development, suggesting that the CSF contained in the ventricles may be a source of extracellular taurine in the developing cortex [93]. Indeed, the injection of taurine into the AF rescued the cortical phenotypes of TauT KO embryos [93].

In summary, extracellular taurine functions as an agonist for GABA_A_ receptors, GABA_B_ receptors, and glycine receptors. Extracellular taurine is released from the cells via volume-sensitive channels or is present in CSF, which originates from AF in the initial phase of brain development, contained in the ventricles of the developing brains.

#### 3.2.2. Other Mechanisms Related to Various Physiological Functions of Taurine during Development

Taurine possesses various cellular and physiological functions [9]. Taurine functions as a membrane stabilizer [105], is involved in cell volume regulation [9,106] and cellular calcium homeostasis [84,107], is incorporated into modified uridines in mitochondrial tRNAs [108,109,110,111], exhibits robust antioxidant effects [9,112,113,114], modulates inflammation and apoptosis [9,115,116,117], and promotes tissue repair in combination with branched-chain amino acids [118]. These basic but vital functions of taurine at the cellular level must support normal systemic development. Some of the various phenotypes observed in taurine depletion experiments could be related to these principal cellular functions, although further studies are needed from such a point of view.

#### 3.2.3. Function of Taurine as a Factor to Shape the Gut Microbiota

In the last division of this section, I will discuss a lesser acknowledged although potentially critical role of taurine. The colonization of gut microbiota in the offspring relies on the maternal resident flora as a primary source of the microbiome during perinatal periods [119]. Microbes colonize human and other mammalian bodies during the first moments of life and coexist with the host throughout later life [120]. Many lines of studies have demonstrated that gut microbiota play significant roles in the regulation of the physiology of the hosts [121]. The gut microorganisms that reside in the host during development can affect the overall development of the host [119,122,123].

Bile acids (BAs) are the multifunctional products of cholesterol metabolism that occur in a wide range of vertebrates and aid in the absorption of fats and fat-soluble vitamins from the diet [119,124,125]. BAs are synthesized in the hepatocytes of the liver and secreted into bile. Most BAs are actively absorbed by the ileum and enter the portal vein [124]. After their synthesis in the hepatocytes, primary BAs are conjugated by N-acylamidation with taurine or a taurine-derivative or, less commonly, with glycine in rodents [124]. After conjugated BAs are secreted into the intestinal tract, they are modified by the gut microbiota to produce secondary BAs, which lack taurine or glycine residues [119,126]. The synthesis of BAs is subject to negative feedback control, which is modulated by the nuclear receptor farnesoid X receptor (FXR) in the ileum and liver [127]. By comparing BA profiles in germ-free (GF) mice and in conventionally raised (CONV-R) mice, Sayin demonstrated that the gut microbiota not only regulated the secondary bile acid metabolism but also modified bile acid synthesis in the liver by modulating FXR signaling [127]. The gut microbiota modulate BA synthesis by regulating the expression of the enzymes in hepatocytes involved in BA synthesis [119,128]. In other words, BAs synthesized by the host influence intestinal bacterial compositions, and intestinal bacteria conversely modify the circulating BA composition in the host [119,128]. Notably, Sayin et al. also found that taurine levels in the liver decreased in CONV-R mice than in GF mice, while the expression levels of CSAD and TauT increased in the livers of CONV-R mice compared to those of GF mice [127]. Although the detailed underlying mechanisms for enhancing hepatic taurine biosynthesis and transport in CONV-R mice remain unknown, it can be at least inferred that the homeostatic mechanism for taurine may be involved in gut microbiota–BA interactions.

Miyazaki et al. analyzed BA profiles in taurine-depleted cats and demonstrated that the total BA concentration in bile was higher in the control than that in the taurine-depleted group [129]. However, the total BA concentration in serum was lower in the control than that in taurine-depleted cats. BA profiles in bile also differed between control and taurine-depleted cats [129]. The authors explained the mechanism as follows. Impaired BA metabolism in the liver is induced by decreased mitochondrial cholesterol 27-hydroxylase expression and mitochondrial activity [129]; taurine depletion decreases taurine-modified mt-tRNAs, resulting in reduced expressions of CYP27A1, a key enzyme for primary BA synthesis. BA synthesis, particularly in chenodeoxycholic acid synthesis pathway, decreases [129]; in addition, BA excretion into bile is decreased, and, conversely, excretion into the peripheral circulation is increased [129]. These results illustrate the function of taurine in BA homeostasis. Recently, Stacy et al. revealed a taurine-based mechanism in which BAs influence gut microbiota, resulting in enhanced resistance to pathogens [130,131]. The authors found that the gut microbiota from previously infected hosts displays enhanced the resistance to infections. This long-term functional remodeling of the host’s immunity is associated with altered BA metabolism resulting in the expansion of bacterial taxa that utilize taurine [130]. Interestingly, supplying exogenous taurine alone is sufficient for inducing alterations in microbiota function and enhancing resistance to the pathogens [130]. Further analyses showed that taurine potentiates microbial production of sulfide, an inhibitor of cellular respiration, which plays a protective role against the invasion of the host by numerous pathogens [130]. This suggests that taurine sustains and trains the microbiota, which promotes host resistance to subsequent infection [130].

In summary, taurine can directly influence the microbiome by its potential as a sulfide supplier or by its ability to conjugate with primary BAs. Therefore, taurine could indirectly affect the development of the host by shaping resident microbiota. Such functions should be addressed in future studies.

### 3.3. Possible Influence of Limited Levels of Taurine during Development on Disease Risk in Adults

Low birth weight relates closely to the increased incidence of coronary heart disease and related disorders, such as stroke, hypertension, and adult-onset diabetes [132]. A new ‘developmental’ hypothesis for the etiology considers specifically how development in early life affects the development of chronic diseases later in life [132,133]. The theory was proposed by Barker and now developed to the concepts of *developmental programming* or *the developmental origins of the health and disease hypothesis (DOHaD)* [134,135]. According to the hypothesis, exposure in early life to environmental factors, such as maternal nutrition, infant feeding methods, maternal stress, and infection, influences the long-term risk of various diseases [135,136]. Therefore, IUGR exerts its long-term effects on disease susceptibility later in life [42,137]. As discussed earlier in this review, IUGR is associated with reduced TauT activity, and, importantly, low plasma concentrations of taurine are often found in IUGR fetuses [42,46]. Reduced placental TauT activity is associated with maternal obesity and PE [47,48]. Similarly, IUGR is often associated with maternal obesity and PE [138,139]. Furthermore, maternal nutrient restriction reduced placental TauT expression and the concentration of amino acids, including taurine, which restricted the growth of the fetus [49]. These results suggest that reduced taurine levels, induced by, among other factors, maternal obesity, PE, and malnutrition during fetal and infant development can result in limited body growth and could increase the risks of various chronic diseases in later life. Future studies are strongly needed from such a point of view.

## 4. Conclusions

**Figure 3:**
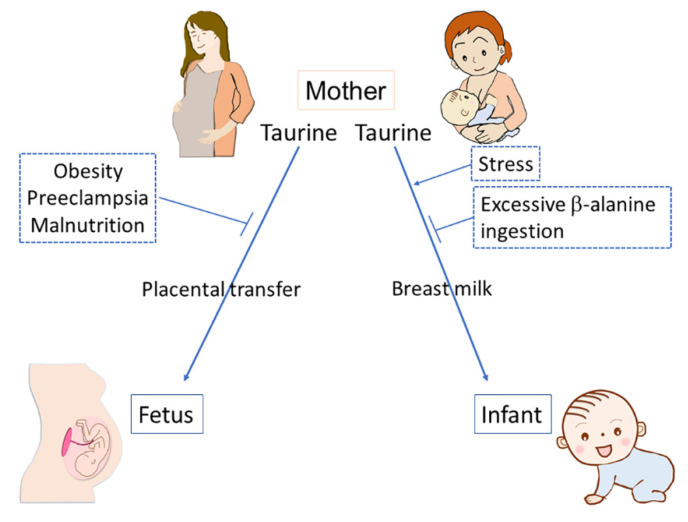
Taurine transfers from mother to offspring via either the placenta or breast milk and several environmental factors affect the transfer of taurine. Maternal obesity, preeclampsia, and malnutrition can inhibit the placental transfer of taurine. Excessive β-alanine ingestion can inhibit taurine transfer via breast milk, while maternal stress can enhance it.

Taurine transport from mother to offspring underlies the functioning of various physiological factors that determine healthy development. Taurine transfer via the placenta can be inhibited by various environmental factors, including maternal obesity, PE, and malnutrition. In breast milk, maternal stress increases taurine concentration, while excessive maternal β-alanine ingestion results in a decrease in the concentration of taurine (Figure 3). Taurine depletion has diverse adverse effects on offspring development; further studies are needed concerning the regulatory mechanisms of taurine transport, the environmental factors that influence the activity of taurine transport, and the immediate and long-term health outcomes associated with limited taurine availability during prenatal and perinatal periods.
